# CXCL9, CXCL10, and CCL19 synergistically recruit T lymphocytes to skin in lichen planus

**DOI:** 10.1172/jci.insight.179899

**Published:** 2024-10-22

**Authors:** Anna E. Kersh, Satish Sati, Jianhe Huang, Christina Murphy, Olivia Ahart, Thomas H. Leung

**Affiliations:** 1 Department of Dermatology, University of Pennsylvania School of Medicine, Philadelphia, Pennsylvania, USA.; 2 Corporal Michael Crescenz Veterans Affairs Medical Center, Philadelphia, Pennsylvania, USA.

**Keywords:** Dermatology, Cytokines, Skin, T cells

## Abstract

Lichen planus (LP) is a chronic, debilitating, inflammatory disease of the skin and mucous membranes that affects 1%–2% of Americans. Its molecular pathogenesis remains poorly understood, and there are no FDA-approved treatments. We performed single-cell RNA sequencing on paired blood and skin samples (lesional and nonlesional tissue) from 7 patients with LP. We discovered that LP keratinocytes and fibroblasts specifically secrete a combination of CXCL9, CXCL10, and CCL19 cytokines. Using an in vitro migration assay with primary human T cells, we demonstrated that CCL19 in combination with either of the other 2 cytokines synergistically enhanced recruitment of CD8^+^ T cells more than any individual cytokine. Moreover, exhausted T cells in lesional LP skin secreted CXCL13, which, along with CCL19, also enhanced recruitment of T cells, suggesting a feed-forward loop in LP. Finally, LP blood revealed decreased circulating naive CD8^+^ T cells compared with that in healthy volunteers, consistent with recruitment to skin. Molecular analysis of LP skin and blood samples increased our understanding of disease pathogenesis and identified CCL19 as a new therapeutic target for treatment.

## Abstract 2

Lichen planus is an inflammatory T cell mediated skin disease of unknown etiology. Combination of skin-secreted CXCL9, CXCL10, and CCL19 cytokines amplified T-cell recruitment.

## Graphical abstract

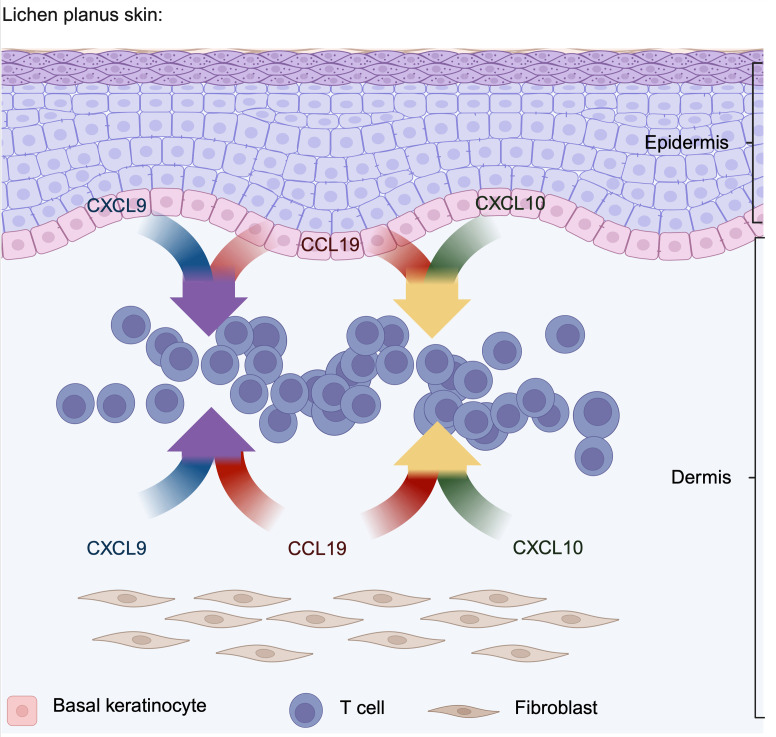

## Introduction

Lichen planus (LP) is a chronic, inflammatory disease of unknown etiology. It is characterized by pruritic, purple, polygonal papules that affect the skin, oral and/or genital mucosa, hair, and nails (1). LP affects the quality of life of 1%–2% of Americans. Although this incidence rate is similar to that of psoriasis (3%), we have a more limited understanding of disease pathogenesis, and there are no FDA-approved treatments. It remains an area of unmet clinical need.

LP is histologically characterized by a robust, band-like infiltrate of lymphocytes in the skin and epidermal keratinocyte apoptosis. Infiltrating lymphocytes are predominantly polyclonal or oligoclonal CD8^+^ T cells (2–4). Prior global transcriptomic profiling of patient samples identified a type II IFN inflammatory response (5). IFN-γ stimulated skin keratinocytes to express MHC-I and increase their susceptibility to cytotoxic CD8^+^ T cells. An unresolved question was the cellular source of IFN-γ.

IFN-γ was shown to induce production of CXCL9 and CXCL10 chemokines in epidermal keratinocytes, and CXCR3 (the receptor for CXCL9 and CXCL10) was previously identified on infiltrating CD4^+^ and CD8^+^ T cells in LP (6–9). Thus, CXCL9 and CXCL10 may aid in T cell recruitment into the skin. However, expression of CXCL9 and CXCL10 by epidermal keratinocytes is not unique to LP. Previous work in vitiligo, an autoimmune disease characterized by CD8^+^ T cells targeting melanocytes, demonstrated an essential role for CXCL9 and CXCL10 in the recruitment of CD8^+^ T cells to skin (10, 11). Targeted elimination of these chemokines resulted in improvement in clinical disease. While vitiligo and LP are clinically and histologically distinct T cell–mediated diseases, we sought to improve our understanding of LP pathogenesis by exploring the role of chemokines in T cell recruitment to the skin.

We discovered that LP fibroblasts and basal keratinocytes specifically secrete CCL19, CXCL9, and CXCL10. We demonstrate in vitro that this combination of chemokines can powerfully and synergistically induce T cell migration, and we conclude this chemokine milieu is important for T cell recruitment to the skin in LP.

## Results

### Immune cell landscape in LP skin consists predominantly of CD8^+^ T cells.

**Figure 1:**
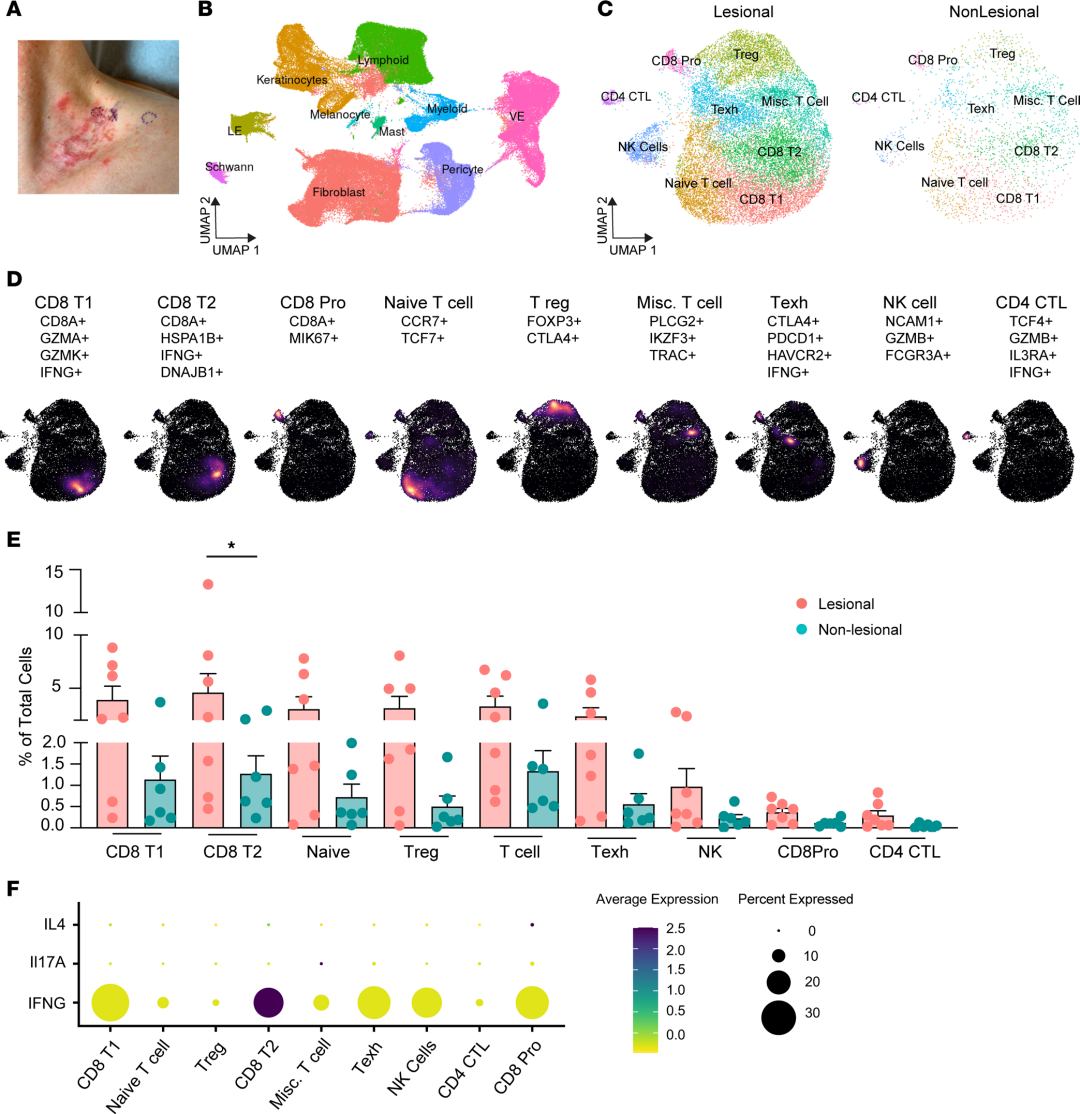
Immune cell landscape in lichen planus. (**A**) Clinical photograph of lesional and nonlesional skin biopsies from a patient with lichen planus (LP). (**B**) Identification of cell clusters from lesional and nonlesional LP skin (*n* = 13). (**C**) UMAP depicting subclustering of lymphoid cells. (**D**) Analysis of individual subclusters. Marker genes are shown. Density plots demonstrate location of subgroup within UMAP. (**E**) Bar plots showing relative contribution as a percentage of total cells. *P < 0.05, 1-way ANOVA. (**F**) Dot plot demonstrating levels and percent of cells expressing *IFNG, IL17A,* and *IL4*. Data are shown as the mean ± SEM.

We collected 4-millimeter skin biopsies from lesional and nonlesional skin from 7 patients with LP (Figure 1A and Supplemental Table 1; supplemental material available online with this article; https://doi.org/10.1172/jci.insight.179899DS1). Three male and 4 female patients were included, with a median age of 62 years (mean, 57 years). All patients had clinically active disease; 5 patients were not receiving treatment, and 2 patients were on oral treatment (hydroxychloroquine and prednisone, respectively). We generated 188,607 high-quality single-cell RNA-sequencing (scRNA-Seq) profiles (Supplemental Table 2). Unsupervised cell clustering of scRNA-Seq profiles revealed 29 unique cell populations that were annotated to 10 cell types using marker gene identification and mapping to single-cell databases (Figure 1B and Supplemental Figure 1, A–E). The cell types were shared between lesional and nonlesional skin samples, and lymphoid cell populations were consistently enriched by number in all lesional LP skin samples (Supplemental Figure 1, B and C).

Next, we subclustered the lymphoid cell population and identified 9 cell types, 8 T cell subtypes and 1 NK cell population (Figure 1, C and D, and Supplemental Figure 1, F and G). Consistent with prior studies, CD8^+^ T cells were the predominant type. Three populations of CD8^+^ T cells were identified: CD8^+^ T1 (*CD8A*, *GZMA*, *GZMK*, *IFNG*), CD8^+^ T2 (*CD8A*, *IFNG*, *HSPA1A*, *DNAJB1*), and a proliferating CD8^+^ population “CD8^+^ Pro” (*MKI67*, *CD8A*). As a percentage of total lymphoid infiltrate, the CD8^+^ T2 population was significantly increased in LP skin (*P* = 0.04, Figure 1E). This population of T cells was notable in that it expressed the highest levels of IFN-γ (*IFNG*) and granzyme A (Figure 1F and Supplemental Figure 1I). As a percentage of lymphoid cell infiltrate, we did not see major differences in most subpopulations, suggesting increased recruitment of all T cells. Notably, we found an increased percentage of Tregs (*FOXP3*, *CTLA4*) in LP skin (*P* = 0.02) (Supplemental Figure 1G), similar to other inflammatory skin diseases (12). Three additional populations of T cells were identified that consisted of both CD4^+^ and CD8^+^ T cells, including naive (*CCR7*, *TCF7*), an exhausted T cell phenotype “Texh” (*CTLA4*, *PDCD1*, *HAVCR2*, *IFNG*), and a miscellaneous T cell category (*TRAC*, *IKZF3*, *PLCG2*) (Figure 1D). Exhausted T cells were characterized by their expression of PD1 (*PDCD1*), low levels of cytokine production (i.e., IFN-γ), and high levels of *CXCL13* expression (13, 14). CD4^+^ cytotoxic T lymphocytes (CTLs) (*TCF4*, *IL3RA*, *IFNG*, *GZMB*) represented a small population of cells in lesional LP skin. Finally, a few prior studies have suggested a role for Th17 cells in LP (15–17). However, we observed no significant *IL17A* production in our T cell populations (Figure 1F). Taken together, our data demonstrated that CD8^+^ T2 cells were the major source of IFN-γ (Figure 1F).

### LP skin secretes CXCL9, CXCL10, and CCL19 cytokines.

**Figure 2:**
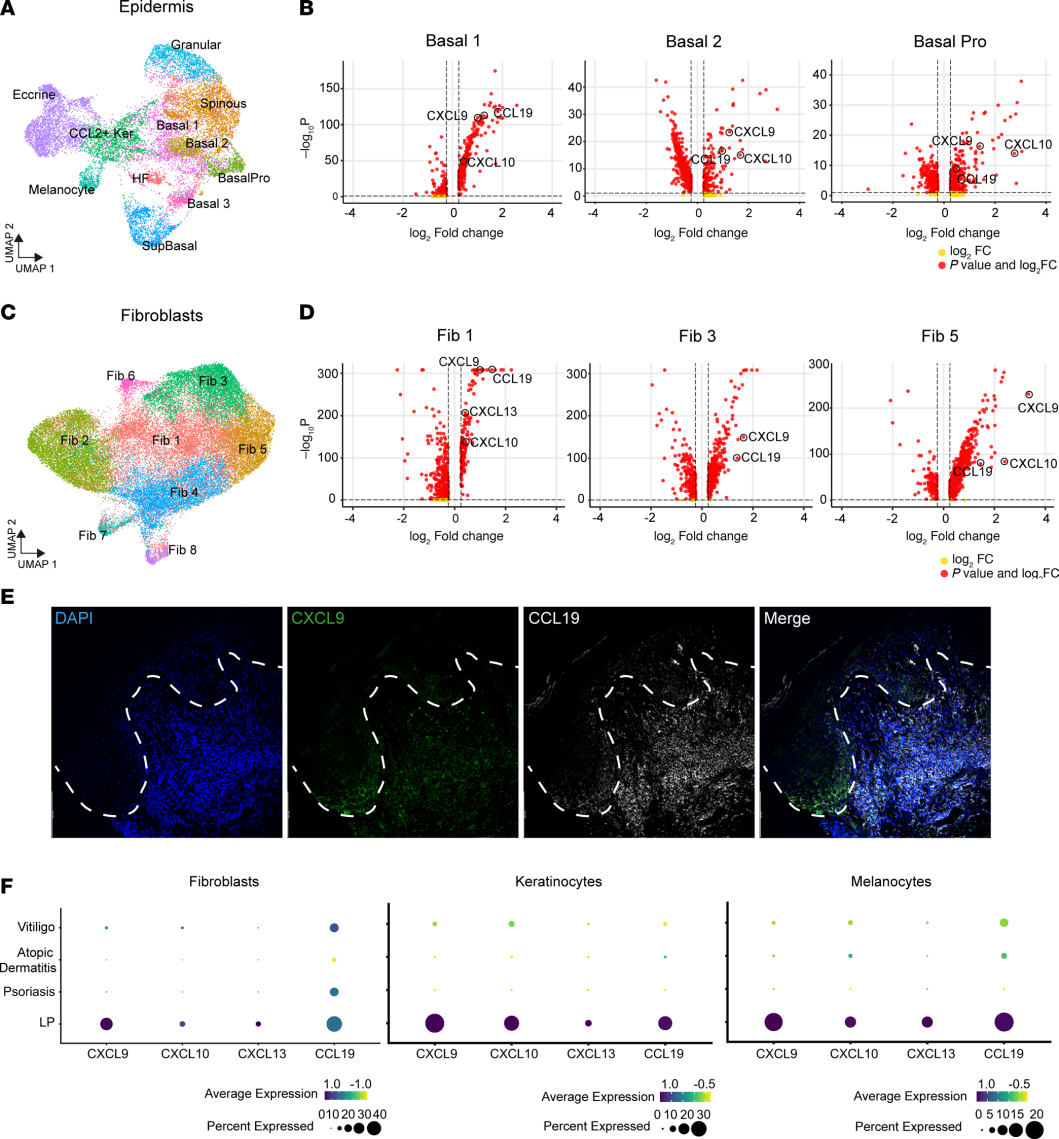
Lichen planus skin secretes CXCL9, CXCL10, and CCL19. (**A**) UMAP depicting subclustering of epidermal skin cells. (**B**) Volcano plots of differential gene expression from basal keratinocyte subpopulations from lichen planus (LP) lesional versus nonlesional skin. Expression of *CXCL9*, *CXCL10*, and *CCL19* is labeled on the plots. A *P* value of less than 0.01 (Wilcox’s test) and a 2-fold expression change was used for significance. (**C**) UMAP depicting subclustering of fibroblasts. (**D**) Volcano plots of differential gene expression from fibroblast subpopulations from LP lesional versus nonlesional skin. Expression of *CXCL9*, *CXCL10*, and *CCL19* is labeled on the plots. A *P* value of less than 0.01 (Wilcox’s test) and a 2-fold expression change was used for significance. (**E**) Representative immunofluorescence images depicting localization of CXCL9 (green), CCL19, (white), and DAPI (blue) in LP skin (*n* = 5 patient samples). The white dotted line depicts the epidermal-dermal junction. Scale bars: 100 μm. (**F**) scRNA-Seq data from publicly available single-cell datasets for vitiligo, psoriasis, and atopic dermatitis were used to analyze skin cells for their expression of chemokines. Dot size corresponds to percentages of cells expressing chemokine, while color corresponds to level of gene expression.

To assess how LP skin may recruit lymphoid cell populations, we subclustered epidermal cell populations and identified eleven unique clusters, which were annotated based on expression of canonical marker genes (Figure 2A and Supplemental Figure 2, A and B). We identified 4 populations of basal cells along with suprabasal cells, melanocytes, and cells of the hair follicle and eccrine glands (18, 19). LP is histologically characterized by apoptosis in the basal layer of keratinocytes. Within basal keratinocytes, *CXCL9* and *CXCL10* were induced as much as 8-fold in lesional skin compared with nonlesional skin, making them two of the highest induced genes (Figure 2B and Supplemental Figure 3A). Melanocytes also markedly expressed *CXCL9*, *CXCL10*, and *CCL19* (all 2-fold) compared with nonlesional skin (Supplemental Figure 3A). Transcription factor analysis of the keratinocyte populations showed an upregulation of IRF7, ETV7, and STAT2 (Supplemental Figure 3B).

Fibroblasts subclustered into 8 unique populations, and 4 subpopulations exhibited increased expression of *CXCL9* (up to 10-fold) and *CXCL10* (up to 5-fold) in LP lesional skin compared with nonlesional skin (Figure 2, C and D). Interestingly, all fibroblast populations also substantially induced expression of *CCL19* (average 3-fold induction) (Figure 2, D and E). CCL19 is a chemokine typically expressed in thymus and lymph nodes to regulate immune cell trafficking, but it does not have an established role in skin inflammatory diseases (20). One population of basal keratinocytes, Basal 1, also induced *CCL19* significantly (Figure 2B). Transcription factor analysis of these fibroblast clusters showed upregulation of IRF7, STAT1, STAT2, and RUNX3 (Supplemental Figure 3D).

To confirm our scRNA-Seq findings, we performed immunohistochemistry on LP skin biopsies. CXCL9 and CXCL10 were primarily expressed by keratinocytes in the lower layers of the epidermis and fibroblasts in the superficial dermis (Figure 2E). CCL19 was also strongly expressed in the lower levels of epidermis and in the superficial dermis. We confirmed that CCL19^+^ dermal staining came from fibroblasts (CD3^–^, vimentin^+^, CCL19^+^) and to a lesser extent tissue-infiltrating T cells (Supplemental Figure 4A). We additionally assessed skin biopsies of patients with lichen planopilaris (LPP) and psoriasis. LPP is a scalp-restricted clinical variant of LP. Consistent with prior reports, we found increased expression of CXCL10 and CCL19 that localized to the hair follicle epithelium (Supplemental Figure 4B) (21). We did not observe staining for these chemokines in psoriasis skin samples (Supplemental Figure 4C). Taken together, CXCL9, CXCL10, and CCL19 were the major cytokines induced in keratinocytes and fibroblasts of LP skin.

Next, we compared the chemokine environment in LP to that in other T cell–mediated skin diseases. We analyzed publicly available scRNA-Seq skin datasets for psoriasis, atopic dermatitis, and vitiligo (22–24). Within fibroblasts, keratinocytes, and melanocytes, we found that LP lesional skin had the strongest expression of *CXCL9*, *CXCL10*, and *CCL19* as well as the highest frequency of fibroblasts expressing these chemokines (Figure 2F). Thus, LP skin strongly secretes a unique combination of CXCL9, CXCL10, and CCL19 cytokines.

**Figure 3:**
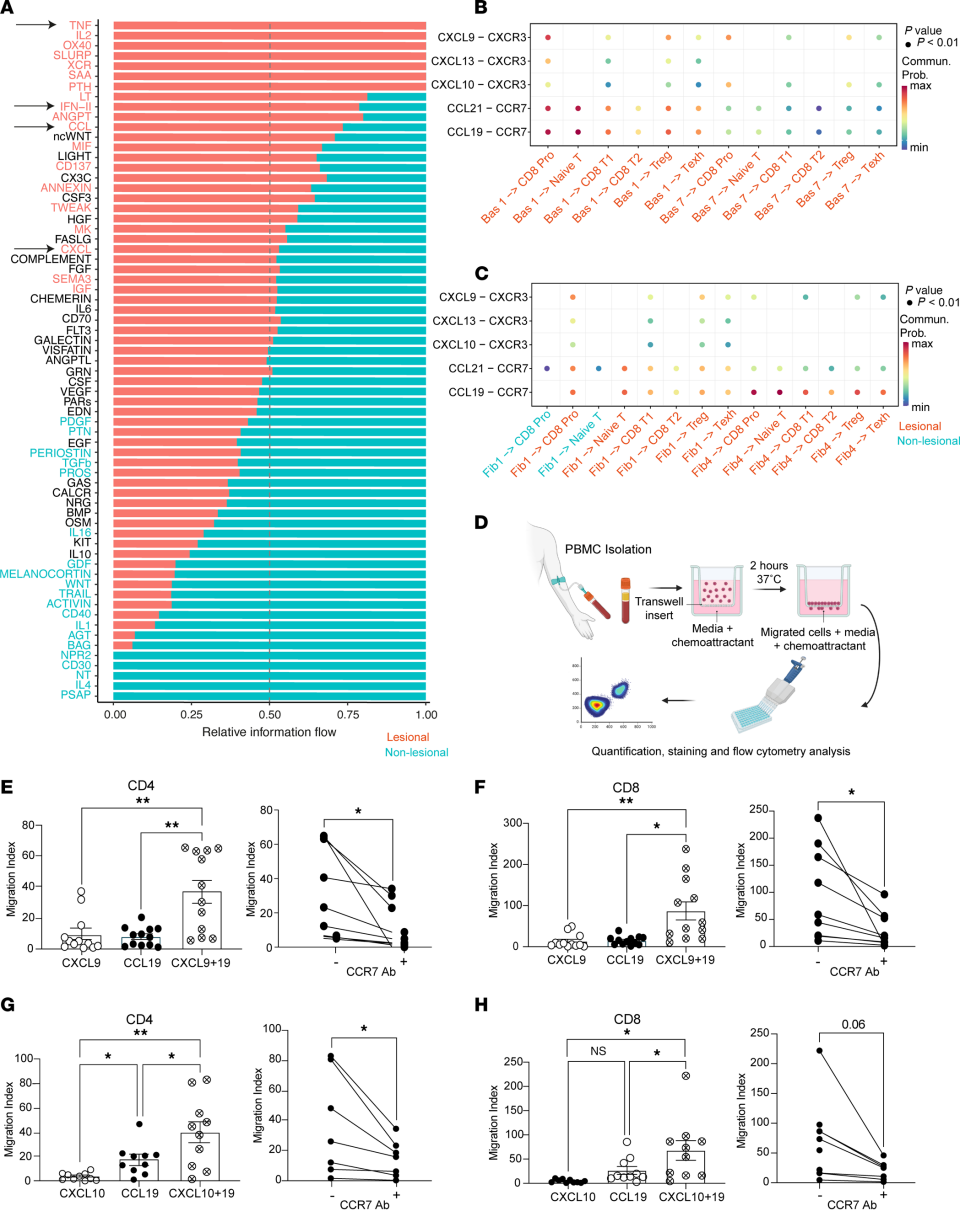
CCL19 synergizes with skin-secreted CXCL9 or CXCL10 to recruit T cells. (**A**) Global analysis of ligand-receptor pathways. Arrows highlight relevant signaling. (**B**) Analysis of cell-to-cell interactions between epidermal keratinocytes cells and immune cells in lichen planus (LP) lesional skin. Dot color illustrates communication probability, and dot size illustrates *P* value. (**C**) Analysis of cell-to-cell interactions between dermal fibroblasts and immune cells in lesional (salmon color) and nonlesional (blue color) LP skin. Dot color illustrates communication probability, and dot size illustrates *P* value. (**D**) Schematic of migration assay. (**E**–**H**) Left: migration index (no. of migrated cells in response to cytokine/no. of migrated cells in response to control) for CD4^+^ and CD8^+^ T cells in response to different conditions of CXCL9, CXCL10, and CCL19 (*n* = 12). Right: Combined treatment with or without CCR7 blocking antibodies (*n* = 9). Data are shown as the mean ± SEM. *P < 0.05, **P < 0.01, 1-way ANOVA was used for migration assays, and 2-tailed paired Student’s *t* test was used for CCR7 antibody analysis.

CellChat computationally identifies potential ligand-receptor interactions within a population of cells. Global analysis of lesional and nonlesional skin from patients with LP revealed upregulation of signaling pathways in the cytotoxic and Th1 lymphocyte response (TNF, IL2, OX40, LT, IFN-II) and chemokine signaling (CCL, CXCL) (Figure 3A and Supplemental Figure 5, A and B). In lesional skin, basal keratinocytes and fibroblasts secreted CXCL9 and CXCL10, which were received by CXCR3 expressed on CD8^+^ Pro T cells, CD8^+^ T1 cells, Tregs, and Texh populations in lesional LP skin only (Figure 3B). CCL19 expressed by fibroblasts and basal keratinocytes was received by CCR7 expressed on CD8^+^ Pro T cells, naive T cells, CD8^+^ T1 cells, CD8^+^ T2 cells, Tregs, and Texh cells (Figure 3C). Taken together, CellChat analysis suggests that CXCL9, CXCL10, and CCL19 signals converge on T cells in LP skin.

### CCL19 works synergistically with CXCL9 and CXCL10 to recruit T cells.

To test whether these cytokines recruit T cells, we performed in vitro migration assays with PBMCs from healthy donors (Figure 3D). We quantified migration of CD4^+^ and CD8^+^ T cells by flow cytometry (Supplemental Figure 6A). We tested migration in response to CXCL9, CXCL10, or CCL19 as well as combinations of both CXCL9 and CCL19 and CXCL10 and CCL19 (Figure 3, D–H). The migration index is the ratio of cells that migrated in response to a chemokine stimulus divided by the number of cells that migrated in response to control media.

Compared with vehicle control, CD4^+^ T cells exhibited more migration toward CXCL9 (mean migration index, 9.5 ± 3.6) and CCL19 (mean migration index, 8.1 ± 1.8) alone (Figure 3E, left). Combination treatment with CXCL9 and CCL19 induced CD4^+^ T cells to migrate significantly more strongly (mean migration index, 36.7 ± 7.3). In fact, these cells migrated more than double the sum of the individual chemokine migration indices, suggesting a synergistic response. Blocking antibodies against CCR7, the receptor for CCL19, significantly ameliorated the combined treatment’s effect (mean migration index, 12.3 ± 4.5) (Figure 3E, right).

CD8^+^ T cells showed an even more pronounced response (Figure 3F). Combination treatment with CXCL9 and CCL19 induced CD8^+^ T cells to migrate significantly more strongly (mean migration index, 87.0 ± 21.9) than the sum of individual chemokine migration indices for CXCL9 (mean migration index, 13.4 ± 4.9) and CCL19 (mean migration index, 15.0 ± 3.1). Blocking antibodies against CCR7 also reduced the combined treatment’s effect (mean migration index, 29.0 ± 10.8) (Figure 3F, right). Taken together, combination treatment of CXCL9 and CCL19 was synergistic for T cell migration and induced almost 3-fold more CD8^+^ T cells migration compared with CD4^+^ T cells.

We repeated migration assays with CXCL10 with and without CCL19. CD4^+^ T cells demonstrated increased migration toward CXCL10 (mean migration index, 3.8 ± 1.0) and CCL19 (mean migration index, 17.5 ± 4.0) (Figure 3G). Combination treatment also induced CD4^+^ T cells to migrate more (mean migration index, 40.0 ± 9.0) than the sum of the migration indices of each individual chemokine. CCR7 blocking antibodies ameliorated this effect (mean migration index, 14.8 ± 4.6) (Figure 3G, right). CD8^+^ T cells again showed a more pronounced response (Figure 3H). Combination treatment with CXCL10 and CCL19 induced CD8^+^ T cells to migrate more strongly (mean migration index, 68.0 ± 20.3) than the sum of the migration indices for each individual chemokine for CXCL10 (mean migration index, 4.7 ± 1.1) and CCL19 (mean migration index, 26.8 ± 8.1). CCR7 blocking antibodies also reduced this effect (mean migration index, 20.8 ± 5.9), but it did not reach statistical significance (*P* = 0.06; Figure 3H, right panel). In summary, CCL19 worked with CXCL9 or CXCL10 to synergistically amplify the migration of CD4^+^ and CD8^+^ T cells. This effect was greater on CD8^+^ T cells than CD4^+^ T cells.

### T cell–secreted CXCL13 synergizes with CCL19 to recruit CD8^+^ T lymphocytes.

**Figure 4:**
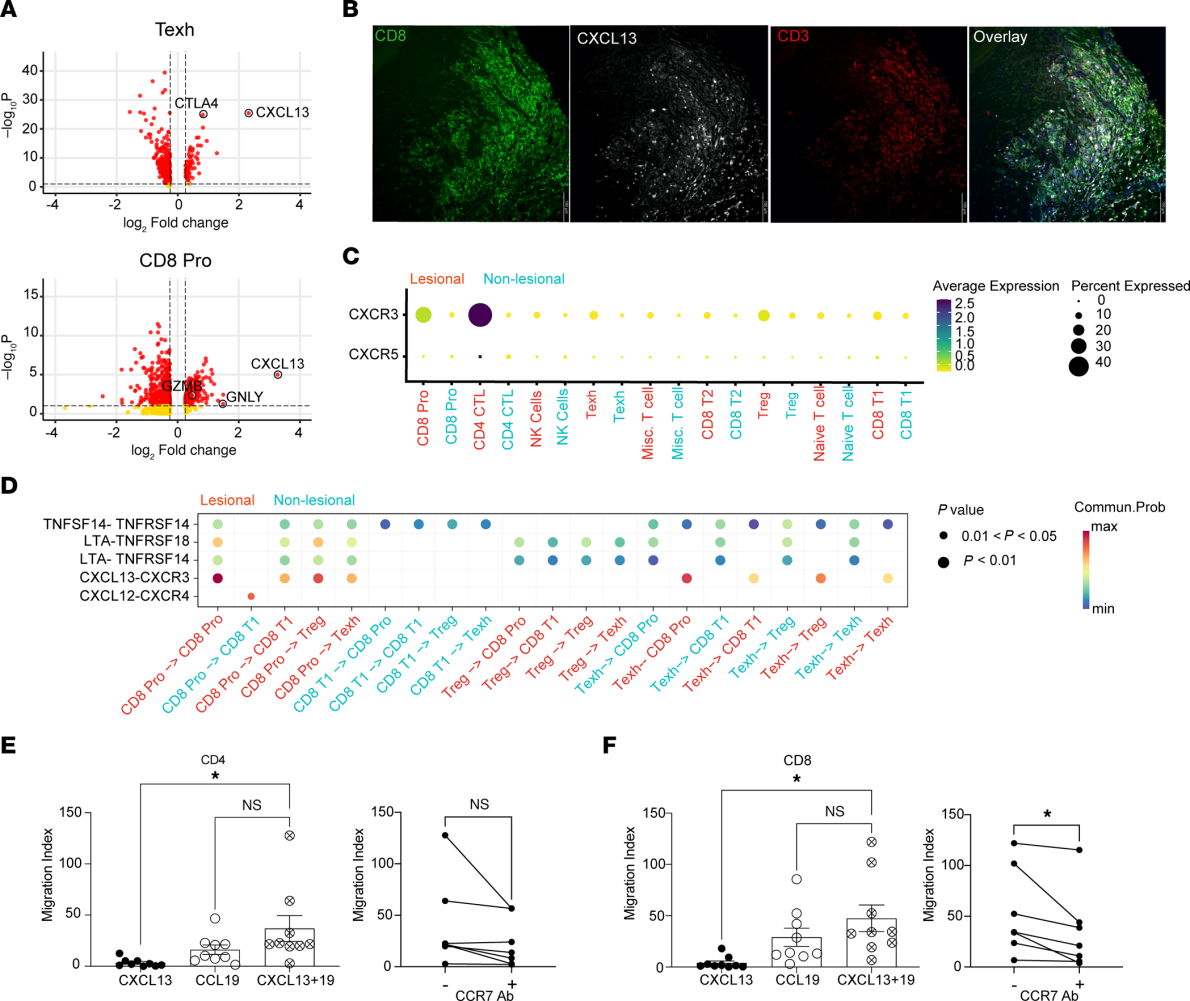
CCL19 synergizes with T cell–secreted CXCL13 to recruit T cells. (**A**) Volcano plots of differential gene expression from exhausted (Texh) and CD8^+^ proliferating (CD8^+^ Pro) T cell populations from lesional versus nonlesional lichen planus (LP) skin. Expression of *CXCL13*, *CTLA4*, *GZMB*, and *GNLY* is labeled. (**B**) Representative immunofluorescence images of LP skin depicting localization of CD4 (green), CXCL13 (white), CD3 (red), and DAPI (blue) (*n* = 5 patient samples). Scale bars: 100 μm. (**C**) Dot plot demonstrating levels and percentages of cells expressing *CXCR3* and *CXCR5*. (**D**) Analysis of cell-to-cell interactions between immune cells in lesional (salmon color) and nonlesional (blue color) LP skin. (**E** and **F**) Left: Migration index (no. of migrated cells in response to cytokine/no. of migrated cells in response to control) for CD4^+^ (**E**) and CD8^+^ T cells (**F**) in response to different conditions of CXCL13 and CCL19 (*n* = 9). Right: Combined treatment with or without CCR7 blocking antibodies (*n* = 7). Data are shown as the mean ± SEM. *P < 0.05, 1-way ANOVA was used for migration assays, and 2-tailed paired Student’s *t* test was used for CCR7 antibody analysis.

We examined our scRNA-Seq dataset to identify other cytokines that may recruit CD8^+^ T cells into LP lesional skin. Prior studies demonstrated that CXCL13 is specifically expressed by exhausted T cells (13, 14, 25). Indeed, *CXCL13* was among the highest induced genes in the Texh (>5-fold induction) and CD8^+^ Pro (>10-fold induction) populations in LP lesional skin compared with nonlesional skin (Figure 4A). Immunohistochemistry of LP skin confirmed that CXCL13 was primarily expressed by infiltrating CD8^+^ T cells (CD3^+^CXCL13^+^CD4^–^) (Figure 4B).

CXCL13 canonically signals through the CXCR5 receptor. However, we did not detect expression of CXCR5 in our dataset. CXCL13 has also been shown to bind CXCR3, and this interaction has been functionally demonstrated to induce T cell migration (26, 27). In our dataset, *CXCR3* was expressed in CD8^+^ Pro, Treg, CD8^+^ T1, and CD4^+^ CTL populations in lesional LP skin (Figure 4C). CellChat analysis highlighted that CD8^+^ Pro T cells may signal to other CD8^+^ Pro T cells in lesional LP skin via CXCL13/CXCR3 interactions as well as CD8^+^ T1 and Treg populations (Figure 4D). Consistently, Texh cells may also signal to CD8^+^ Pro, CD8^+^ T1, and Treg populations (Figure 4D).

We performed migration assays to test the sufficiency of CXCL13 and CCL19 in T cell recruitment. CXCL13 induced migration of both CD4^+^ and CD8^+^ T cells (migration index, 3.3 ± 1.3 and 4.0 ± 2.0, respectively) (Figure 4, E and F). As expected, CCL19 also induced migration of both CD4^+^ and CD8^+^ T cells (migration index, 16.2 ± 4.6 and 29.0 ± 8.9, respectively). Combined treatment significantly increased migration synergistically for both CD4^+^ and CD8^+^ T cells (migration index, 36.9 ± 12.3, *P* = 0.045; 47.5 ± 13.0, *P* = 0.01 respectively) (Figure 4, E and F). Finally, CCR7 blocking antibodies reduced the combined treatment’s migration effect on CD4^+^ and CD8^+^ T cells (migration index, 34.27 ± 14.8 and 23.5 ± 9.0, respectively), but only CD8^+^ was statistically significant.

Taken together, Texh and CD8^+^ Pro T cells in lesional LP skin secrete CXCL13 that synergizes with fibroblast-secreted CCL19 to recruit more CD8^+^ T cells.

### Circulating levels of naive CD8^+^ T cells are decreased in patients with LP.

**Figure 5:**
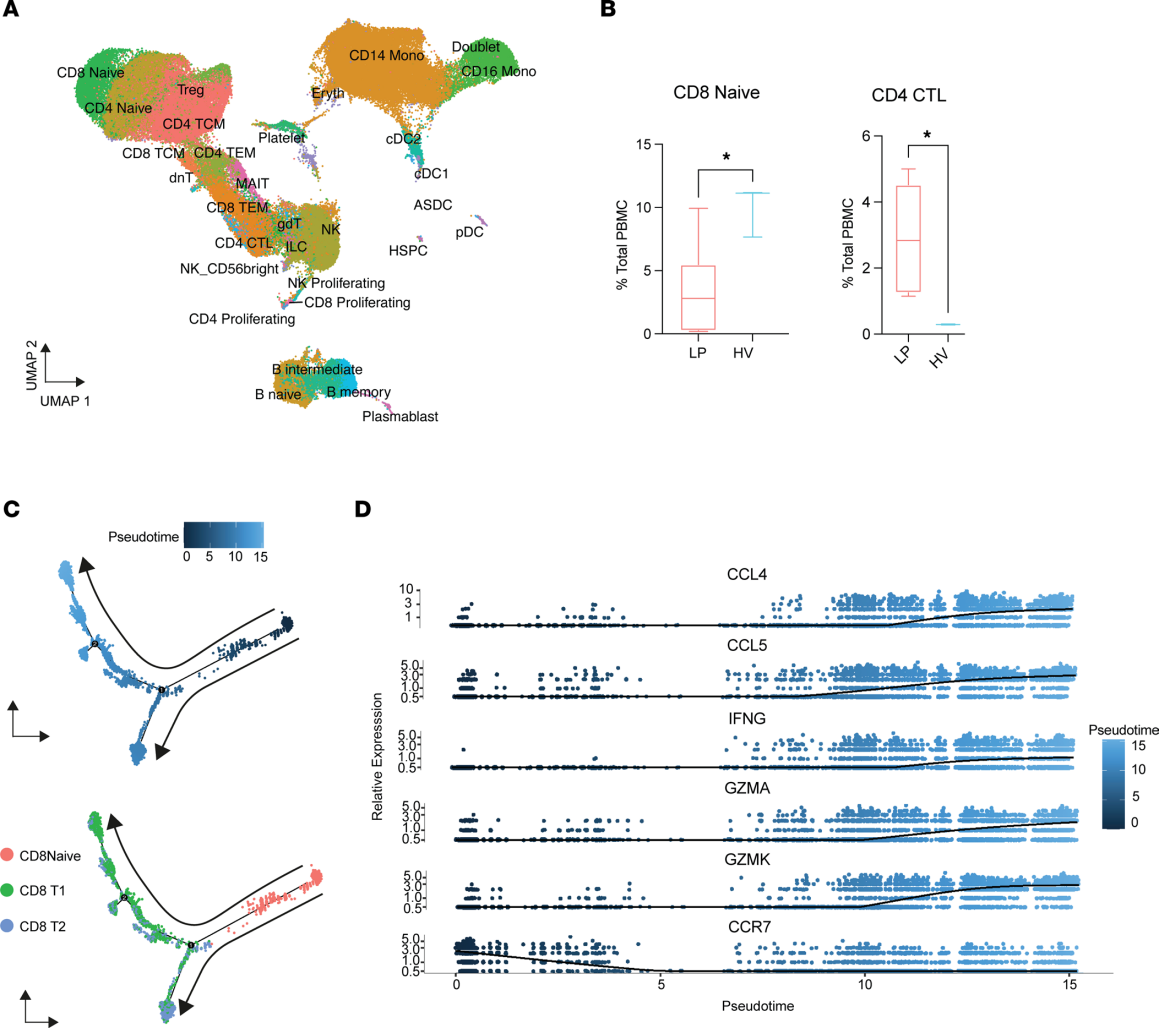
Peripheral naive CD8^+^ T cells migrate to skin in lichen planus. (**A**) Identification of cell clusters from lichen planus (LP) blood (*n* = 7). (**B**) Bar plot showing the relative contribution as a percentage of total cells for CD8^+^ naive and CD4^+^ CTL T cell populations between patients with LP and individuals acting as healthy controls (*n* = 3). Data are shown as the mean ± SEM. *P < 0.05, 2-tailed unpaired Student’s *t* test. (**C**) Pseudotime trajectory of naive CD8^+^ T cells isolated from PBMCs and CD8^+^ T1 and CD8^+^ T2 populations from LP lesional skin. Each dot represents a cell. Top: Trajectory through time (expressed in blue with a pseudotime scale). Bottom: Cells colored according to cell-type origin (naive, salmon; CD8^+^ T1, green; CD8^+^ T2, blue). (**D**) Gene expression changes as the cells progress through the pseudotime trajectory (from naive state in peripheral blood to effector state in tissue).

Tissue-infiltrating lymphocyte populations are generally recruited from peripheral blood. We assessed if there were any changes in circulating immune cell populations between patients with LP and individuals acting as healthy controls. We collected paired PBMC samples from the 7 patients with LP for scRNA-Seq (Supplemental Tables 1 and 2). We used publicly available data for 3 healthy adult controls to create a combined 116,108-cell dataset. After unsupervised clustering, we annotated cell populations using a reference atlas for human PBMCs (Figure 5A and Supplemental Figure 6B) (28, 29). We found two lymphoid populations that changed significantly in frequency between patients with LP and individuals acting as healthy controls. Patients with LP exhibited fewer circulating naive CD8^+^ T cells compared with individuals acting as healthy controls (3.3% ± 1.3% versus 10% ± 1.2% in individuals acting as healthy controls, *P* = 0.02) (Figure 5B). This decrease in circulating naive CD8^+^ T cells may correspond with the influx of CD8^+^ T cells into LP lesional skin. We also observed that CD4^+^ CTLs were enriched 10-fold in circulating blood of patients with LP (2.9% ± 0.7% compared with 0.3% ± 0.01% in individuals acting as healthy controls, *P* = 0.04) (Figure 5B). These cells expressed higher levels of granzyme B compared with those from individuals acting as healthy controls (Supplemental Figure 6C). Notably, none of the circulating cell populations exhibited increased expression of CXCR3 or CCR7 receptors, suggesting that increased recruitment to skin is mediated by changes in cytokine ligand expression in the tissue and not due to changes in receptor expression levels in LP immune cells (Supplemental Figure 6D).

Finally, we performed pseudotime analysis on our LP skin and blood datasets. Pseudotime rationally defines developmental trajectories of analyzed cells based on single-cell transcriptomes that are assumed to be individual variations of developmental states (30). Unbiased analysis of the naive CD8^+^ T cell subset from blood and CD8^+^ T cell subsets from lesional and nonlesional LP skin demonstrated naive peripheral CD8^+^ T cells at the initial state and CD8^+^ T1 and CD8^+^ T2 skin populations at the terminal state (Figure 5C). This trajectory correlated with the upregulation of the genes found on activated CD8^+^ T cells (*IFNG*, *GZMA*, *GZMK*, *CCL4*, and *CCL5*) (Figure 5D). Additionally, the pseudotime trajectory correlated with the downregulation of *CCR7*, which is known to be downregulated after binding to CCL19 (31). Thus, pseudotime analysis suggests that T cell activation in LP is not a systemic finding and only occurs after local recruitment to the skin.

## Discussion

We showed that infiltrating CD8^+^ T cells in LP are the primary source of IFN-γ that may trigger keratinocyte apoptosis (5). Moreover, LP lesional skin secreted a unique combination of CXCL9, CXCL10, and CCL19 chemokines. CCL19 in combination with either CXCL9 or CXCL10 synergistically recruited T cells, especially CD8^+^ T cells. We believe this is a dynamic process by which CCL19 bulk-recruits CCR7-expressing naive T cells to the superficial dermis and that, upon activation, these cells upregulate CXCR3, which strengthens their retention at the dermal epidermal junction. Notably, exhausted T cells and proliferating CD8^+^ T cells in LP skin specifically secreted CXCL13. CCL19 also synergized with CXCL13 to recruit more CD8^+^ T cells, and this mechanism may establish a feed-forward loop.

Individual roles for CXCL9, CXCL10 and CCL19 have been demonstrated in other skin diseases. CCL19-expressing fibroblasts have been identified in atopic dermatitis and localize to leukocyte-rich areas of the skin (23, 32, 33). CXCL9 and CXCL10 are expressed in many other skin diseases, and their functional importance was demonstrated for vitiligo, where inhibition of CXCL10 reduced recruitment of T cells and resulted in skin repigmentation (10, 11, 21). However, compared with these inflammatory skin diseases, LP skin had the strongest expression of all 3 chemokines. There are many different clinical variants of LP; patients may have skin-restricted, mucosa-restricted, or hair follicle–restricted LP (known as LPP or frontal fibrosing alopecia) or involvement of some combination of those sites. Our samples were only obtained from the skin, and similar increased expression of CXCR3 and CCL19 was noted by bulk sequencing in the hair follicle–restricted variant of LP (21). It may be possible that different chemokines recruit T cells to different body sites; a prior study showed increased CCR5 and CXCR3 expression on CD8^+^ T cells in mucosal LP lesions (7). We did not see CCR5 expression in our skin samples. Taken together, we speculate that this specific combination of CXCL9, CXCL10, and CCL19 is responsible for the robust recruitment of all T cells to skin and possibly hair follicles. More studies are needed to assess CCL19 expression and site-specific lymphocyte migration.

Prior work explored whether CCL19 may amplify recruitment of immune cells in other biological contexts. Sezary syndrome is a subtype of cutaneous T cell lymphoma that involves both the skin and blood (34). Combination treatment of CCL19 and CXCL13 enhanced the migration of Sezary CD4^+^ T cells (35). However, CCL19 was not found in skin samples from patients with Sezary syndrome, which may explain why the T cell infiltrate in Sezary syndrome is not as pronounced as in LP. In cancer biology, local production of CCL19 promotes antitumor responses by recruiting more CD8^+^ T cells in lung and ovarian cancer models (36, 37). Thus, CCL19 may also amplify immune cell recruitment in other biological contexts. The robust lymphocytic infiltrate seen in LP has been histologically termed a “lichenoid band reaction,” and this pattern is sometimes seen in patients with allergic drug reactions, lupus erythematosus, and, less frequently, squamous cell carcinoma. It would be interesting to assess the role of CCL19 in the histologically defined lichenoid band reaction.

What stimulates CCL19 production in LP skin? We found induction of STAT1, STAT2, and IRF7 transcription factors in lesional LP keratinocytes and fibroblasts. Prior studies using bacterial or viral infection models demonstrated that CCL19 expression in dendritic cells is similarly driven by activation of STAT1, STAT2, and IRF7 (38). The identification of an initial trigger for LP has remained elusive, and a viral basis for this disease has been speculated by clinicians for decades (39, 40). Future studies are needed to assess the possibility of viral-mediated activation of CCL19 in the skin of patients with LP.

In recent years, the concept that T cells may become “exhausted” after chronic antigen stimulation in tumors and infections has become established. We are intrigued that CXCL13 secreted by exhausted T cells in LP skin also synergizes with CCL19 to recruit T cells. A functional role for exhausted T cells in autoimmune disease remains unexplored, and we posit this feed-forward mechanism may help to sustain LP skin lesions and warrants further exploration.

Finally, we discovered that LP blood contained fewer naive CD8^+^ T cells and more CD4^+^ CTLs. There are no clinical tests available to measure LP disease activity. While cutaneous LP skin activity may be monitored by visual inspection, there are cases of mucosal and esophageal specific lesions that require invasive procedures to assess. A blood test to measure disease activity would benefit patient care by permitting clinicians to assess treatment response. This would help optimize treatment selection and minimize exposure to medication side effects. Future studies should explore whether circulating naive CD8^+^ T cells and/or CD4^+^ CTLs may serve as circulating biomarkers for disease activity.

Molecular analysis of skin and blood improved our understanding of disease pathophysiology. Our data suggest that blocking CCL19 may serve as a novel therapeutic strategy for LP. The ability to directly interrogate diseased skin and blood allows this approach to be generalizable to other systemic inflammatory disorders.

## Methods

### Sex as a biological variable

To address sex as a biological variable, we attempted to recruit equal numbers of male and female patients.

### Demographic information

Patient data and associated demographics are provided in Supplemental Table 1. Demographic information was provided by the participants with options provided by the investigators.

### Human study participants

Patients diagnosed with LP were recruited to the study at the Dermatology Clinic of the Perelman Center for Advanced Medicine at the University of Pennsylvania. Diagnosis in each case had been previously confirmed with histology. Patient demographics are provided in Supplemental Table 1. Four- to 5-millimeter biopsies were taken from lesional and nonlesional skin of patients with active skin disease. All biopsies were transported in saline soaked gauze and processed immediately for optimal cell recovery. Patient whole blood samples were collected in vacutainer tubes with EDTA (Becton Dickinson) to prevent clotting. Healthy volunteer PBMCs and plasma were obtained from Human Immunology Core at the University of Pennsylvania.

### Tissue processing and single-cell gene RNA-seq library preparation and sequencing

Skin biopsies were suspended in serum-free RPMI 1640 media with DNase (0.2 mg/mL, 12633012, Thermo Fisher Scientific), 20 mM HEPES, and 0.25 mg/mL Liberase TM (5401119001, Roche) and minced into <1 mm^3^ pieces using sterile Gradle scissors. The suspension was incubated at 37°C for 90 minutes. The digestion was arrested with FBS and 3 mL of 0.5 M EDTA, and the suspension was subsequently filtered through a 70-micrometer cell strainer (22-363-548, Fisher Scientific). The cells were then washed twice with PBS containing 1% BSA and resuspended in PBS containing 0.04% BSA and taken for counting on a hemocytometer. scRNA-Seq was performed using a 10X Chromium 3 v3.1 kit (1000268, 10X Genomics). The sequencing libraries were prepared per manufacturer’s protocol and sequenced 2 × 100 bp paired-end run on the Illumina HiSeq2000/HiSeq2500 platforms at the BGI America. The raw and processed sequencing data details are given in Supplemental Table 2.

### PBMC and plasma isolation

Patient blood collected in vacutainers was transferred to 50 mL conical tubes and mixed with an equal volume of HBSS (21-023-CV, Corning). The suspension was carefully pipetted over Ficol-paque (17-140-02, GE Healthcare) in a 1:1 ratio by volume. The tubes were centrifuged at 400*g* for 25 minutes with 0 acceleration and 0 deacceleration setting at 10°C. Plasma was carefully aspirated from the top layer, while mononuclear cells were retrieved from the interface. Mononuclear cells were washed with PBS and stored in freezing media (10% DMSO in FBS) in 2 mL cryotube vials. The cryovials were frozen in a freezing container at –80°C for 24 hours before storing in liquid nitrogen.

### Histology and immunohistochemistry

Standard histology and immunostaining protocols were performed, and investigators were blind to tissue origin during histologic staining. In brief, the fresh skin tissue was fixed overnight at 4°C in 4% paraformaldehyde (J19943-K2, Thermo Fisher Scientific). Full thickness skin was removed from the mouse onto a paper towel. The skin was fixed by inverting the paper towel onto the surface of the fixative (4% paraformaldehyde in PBS) and incubated overnight at 4 °C. The following day, the skin was trimmed, placed into tissue cassettes, processed (VIP5b, Sakura) and embedded into wax (Leica Paraplast X-tra) blocks. Blocks were cut using disposable blades (D554P, Sturkey) on a rotary microtome (RM2235, Leica) set at 5 μm thickness. Sections were floated on a water bath (145702, Boekel) set at 43°C and collected onto positively charge glass slides (Fisherbrand Superfrost Plus). Following overnight drying at room temperature, slides were baked for 30 minutes at 60°C, followed by H&E staining using an automated stainer (Leica auto-stainer XL). Slides were processed by the Skin Biology and Disease Resource-Based Core at the Department of Dermatology, University of Pennsylvania, where H&E staining of the slides was performed. H&E-stained sections were examined by a board-certified dermatopathologist under bright-field microscopy. For immunofluorescence microscopy, the following antibodies were used: CD3 (MCA1477, Bio-Rad), CD4^+^ (ab183685, Abcam), CXCL13 (AF801, R&D Biosystems), CXCL9 (ab9720, Abcam), CCL19 (PA5-109488, Thermo Fisher Scientific), and CCL21 (AF366, R&D Biosystems).

### Computational and statistics

#### scRNA-Seq data analysis.

The scRNA sequencing data were mapped to the GRCh38 reference genome to generate gene count and cell barcode matrices using the “cellranger count” function from the cellranger pipeline (version 5.0.1, 10X Genomics). All downstream analysis steps were performed using the R package Seurat (41) (https://github.com/satijalab/seurat/releases/tag/v4.3.0; branch name, version 4.3.0) unless otherwise noted. In brief, seurat functions “Read10X” and “CreateSeuratObject” were used to import and create a merged Seurat object from all filtered feature barcode matrices generated by the cellranger pipeline. Cells with <250 genes, <500 UMI, <0.80 log_10_ genes per UMI and >20% mitochondrial reads were excluded from the merged Seurat object for further analysis. Genes that were detected in less than 10 cells were also discarded. DoubletFinder was used to identify potential cell doublets as a final quality control (42). To determine and regress out the effect of cell cycle, each cell was given a cell cycle phase score using the Seurat function “CellCycleScoring” (43). The data were then log-normalized and scaled by linear regression against the number of reads. The FindVariableFeatures function followed by SelectIntegrationFeatures function (nfeatures = 3,000) was used to identify variable genes from merged Seurat object. For cross-tissue data integration and batch correction, “FindIntegrationAnchors” and “IntegrateData” were applied to the merged Seurat object. Dimensionality reduction was performed using the RunPCA and RunUMAP function generated UMAP plots. Next, Louvain clustering was performed with the “FindClusters” function using the first 40 principal components and at resolution 1.4. We used the ElbowPlot function in Seurat, visual inspection of DimHeatmap plots at different dimensions and R package clustree to choose an optimum number of dimensions and resolution.

#### Cell type annotation.

We used 3 complementary approaches to annotate the identities of different cell clusters. (a) We checked the expression of lineage-specific marker genes identified from previously published scRNA-Seq studies in our query cluster marker genes list and in differentially expressed genes of the query cluster. (b) We applied an unbiased cell type recognition method named deCS (R package) (44), which leverages mapping of the top 100 genes from the query cluster to the reference transcriptomic datasets of known cell types, such as BlueprintEncode (45), MonoccoImmune reference (46), and Database of Immune Cell Expression (DICE) data (47). We first applied deCS to determine if the predicted annotations were consistent with our findings and then assigned the identity to the cluster. (3) For PBMC scRNA-Seq dataset, we annotated our clusters by using Seurat reference mapping function to overlay our gene expression profiles onto the multimodal PBMC atlas. The sample statistics and marker gene dot plots were made by using dittoSeq (v 1.4.1). The uniform manifold approximation and projection (UMAP) was applied to visualize the single-cell transcriptional profile in 2D space based on the SNN graph described above (48). Other bar plots, box plots, violin plots and heatmaps were generated by customized R code through ggplot2 (v3.2.1, R package) (49).

#### Analysis of scRNA-Seq data from other skin diseases.

For additional characterization of skin fibroblasts and keratinocytes, we analyzed previously published datasets for atopic dermatitis, psoriasis, and vitiligo. We downloaded read-level data for the following publicly available datasets: GSE147424 (atopic dermatitis, ref. 23), GSE202011 (psoriasis, ref. 22), and PRJCA006797 (vitiligo, ref. 24).

#### CellChat.

We used R package CellChat (1.5.0) to study the ligand-receptor interaction networks between different immune cell subclusters (50). We performed the ligand receptor interaction analysis on the immune subcluster from the LP scRNA-Seq dataset. The analysis was performed twice, once with all ligand interaction pairs and second on the paracrine signaling network. For our analysis, we considered ligand-receptor interactions that were expressed in at least 10 cells. The CellChat algorithm calculates an aggregated ligand-receptor interaction score base on a method called “trimean.” The CellChat algorithm has the added advantage of comparing two or more single-cell datasets and gives a comparative score for the given cell types. These scores represent the probability of interaction among the ligand-receptor pairs. The probability was then visualized using functions such as netAnalysis_signalingRole_scatter, which visualizes the major sender and receiver across all cell types, and netAnalysis_signalingChanges_scatter, which identifies the major signaling networks acting within a given cell type.

#### Pseudotime.

The single-cell pseudotime trajectory analysis was performed using the Monocle2 R package (v 2.18.0) (51). The LP skin CD8^+^ T cell subclusters were integrated with naive T cell subcluster from PBMC scRNA-Seq data from patients with LP. The integrated object was used as an input for pseudotime analysis, and genes expressed in at least 5 % of the cells were selected to construct the pseudotime trajectory. Following dimensionality reduction using PCA and tSNE method, we ran the densityPeak algorithm to cluster cells based on each cell’s local density (Ρ) and the nearest distance (Δ). Default values were chosen for parameters of the DDRTree method and visualization of dynamically expressed genes along the pseudotime was performed using the “plot_genes_in_pseudotime” function with the default parameters.

### Migration assays

PBMCs isolated from healthy donors were prepared in a single-cell suspension and were allowed to migrate to CXCL9 (392-MG, R&D Biosystems), CXCL10 (266-IP, R&D Biosystems), CXCL13 (801-CX, R&D Biosystems), and CCL19 (361-MI, R&D Biosystems) alone and in various combinations. Chemotaxis assays were performed using 24-well Transwell inserts with 5 μm pore size filters (3421, Corning Costar). After dose-finding experiments were performed, the final concentrations used were 750 ng/mL for CXCL9, 1,000 ng/mL for CXCL10, 1.5 μg/mL for CXCL13, and 750 ng/mL for CCL19. Chemokines were resuspended in 0.6 mL migration medium composed of Iscove medium, 0.5% FBS, and 25 mM HEPES. PBMCs were washed in migration medium and resuspended at 10^7^ cells/mL. 100 μL of cell suspension was placed in upper chamber and allowed to migrate for 2 hours in 37°C. After 2 hours, the migrated cells were processed for quantification of CD4^+^ and CD8^+^ T cells by flow cytometric analysis. Migration results are shown as a migration index, which was calculated by the number of cells migrated in response to chemokine stimulus divided by the number of cells that migrated in response to migration medium alone. For CCR7-blocking antibody experiments, CCR7 antibody (MA5-31992, Thermo Fisher Scientific) was added after resuspension in migration media and allowed to incubate for 15 minutes at 37˚C.

### Flow cytometry analysis

Single-cell suspensions of samples were resuspended in PBS and stained with ZOMBIEGreen viability stain (423112, BioLegend) for 15 minutes in the dark at room temperature. Cells were subsequently stained with the following antibodies suspended in FACS buffer CD3 (UCHT1, 300430, BioLegend), CD4^+^ (RPA-T4, 300559, BioLegend), CD8^+^ (RPA-T8, 301040, BioLegend), CCR7 (G043H7, 353203, BioLegend), and CD19-APC (HIB19, 302211, BioLegend). Dump channel antibodies were CD11b-FITC (ICRF44, 301330, BioLegend), CD11c-FITC (Bu15, 337213, BioLegend), and CD14-FITC (HCD14, 325603, BioLegend). Samples were acquired on a 4-laser BD LSRII flow cytometer, and data were analyzed with FloJo software v10.8.1 (BD).

### Statistics

Presented data have been combined and represent all performed experiments, and unless noted, all experiments were repeated 2–3 times independently. Experiments were not randomized, and investigators were not blinded to allocation during experiments and outcome assessment, unless noted in the text. Comparisons between groups were carried out using 1-way ANOVA (multiple comparisons) and 2-tailed, paired and unpaired Student’s *t* test (as indicated). In all tests, a *P* value of less than 0.05 was considered significant. When appropriate, specific *P* values are provided in figure legends.

### Study approval

Written informed consent was obtained from all participants before enrollment in the study under a protocol approved by the Institutional Review Board of the University of Pennsylvania School of Medicine (IRB #832147). Written informed consent was received for the use of the photographs, and the record of informed consent has been retained.

### Data availability

No unique reagents were generated in the course of this study. All sequencing data have been deposited at Gene Expression Omnibus (GSE254542; https://www.ncbi.nlm.nih.gov/geo/query/acc.cgi?acc=GSE254542). Data values depicted in the main figures as well as the supplemental figures are reported in the Supplemental Data Values file.

This study does not report the original code. Any additional information required to reanalyze the data reported in this paper are available from the corresponding author upon request.

## Author contributions

AEK and THL designed the research studies. AEK conducted the bulk of the experiments with help from SS, CM, and JH on various experiments. SS and AEK performed the analysis of the scRNA-Seq data. AEK analyzed all of the other data. AEK and THL wrote the manuscript, and SS and OA helped with figure preparation.

## Supplementary Material

Supplemental data

Supplemental table 3

Supporting data values

## Version 1 08/27/2024

In-Press Preview

## Version 2 10/22/2024

Electronic publication

## References

[1] Radwan-Oczko M (2018). Psychopathological profile and quality of life of patients with oral lichen planus. J Appl Oral Sci.

[2] Popovska M (2013). T-cell subpopulations in lesions of oral lichen planus. Pril (Makedon Akad Nauk Umet Odd Med Nauki).

[3] Kawamura E (2003). Accumulation of oligoclonal T cells in the infiltrating lymphocytes in oral lichen planus. J Oral Pathol Med.

[4] Gotoh A (2008). Skew in T cell receptor usage with polyclonal expansion in lesions of oral lichen planus without hepatitis C virus infection. Clin Exp Immunol.

[5] Shao S (2019). IFN-γ enhances cell-mediated cytotoxicity against keratinocytes via JAK2/STAT1 in lichen planus. Sci Transl Med.

[6] Ichimura M (2006). Expression profile of chemokines and chemokine receptors in epithelial cell layers of oral lichen planus. J Oral Pathol Med.

[7] Iijima W (2003). Infiltrating CD8+ T cells in oral lichen planus predominantly express CCR5 and CXCR3 and carry respective chemokine ligands RANTES/CCL5 and IP-10/CXCL10 in their cytolytic granules: a potential self-recruiting mechanism. Am J Pathol.

[8] Wenzel J (2009). Type I interferon-associated cytotoxic inflammation in cutaneous lupus erythematosus. Arch Dermatol Res.

[9] Ke Y (2017). Semaphorin4D drives CD8^+^ T-cell lesional trafficking in oral lichen planus via CXCL9/CXCL10 upregulations in oral keratinocytes. J Invest Dermatol.

[10] Rashighi M (2014). CXCL10 is critical for the progression and maintenance of depigmentation in a mouse model of vitiligo. Sci Transl Med.

[11] Richmond JM (2017). Keratinocyte-derived chemokines orchestrate T-cell positioning in the epidermis during vitiligo and may serve as biomarkers of disease. J Invest Dermatol.

[12] Gouirand V (2022). Regulatory T cells and inflammatory mediators in autoimmune disease. J Invest Dermatol.

[13] Thommen DS, Schumacher TN (2018). T cell dysfunction in cancer. Cancer Cell.

[14] Tietscher S (2023). A comprehensive single-cell map of T cell exhaustion-associated immune environments in human breast cancer. Nat Commun.

[15] Schinner J (2023). Skin-infiltrating T cells display distinct inflammatory signatures in lichen planus, bullous pemphigoid and pemphigus vulgaris. Front Immunol.

[16] Schmidt T (2018). T_H_1/T_H_17 cell recognition of desmoglein 3 and bullous pemphigoid antigen 180 in patients with lichen planus. J Allergy Clin Immunol.

[17] Solimani F (2019). Therapeutic targeting of Th17/Tc17 cells leads to clinical improvement of lichen planus. Front Immunol.

[18] Na CH (2019). Integrated transcriptomic and proteomic analysis of human eccrine sweat glands identifies missing and novel proteins. Mol Cell Proteomics.

[19] Wang S (2020). Single cell transcriptomics of human epidermis identifies basal stem cell transition states. Nat Commun.

[20] Gowhari Shabgah A (2022). Does CCL19 act as a double-edged sword in cancer development?. Clin Exp Immunol.

[21] Dubin C (2022). Scalp and serum profiling of frontal fibrosing alopecia reveals scalp immune and fibrosis dysregulation with no systemic involvement. J Am Acad Dermatol.

[22] Castillo RL (2023). Spatial transcriptomics stratifies psoriatic disease severity by emergent cellular ecosystems. Sci Immunol.

[23] He H (2020). Single-cell transcriptome analysis of human skin identifies novel fibroblast subpopulation and enrichment of immune subsets in atopic dermatitis. J Allergy Clin Immunol.

[24] Xu Z (2022). Anatomically distinct fibroblast subsets determine skin autoimmune patterns. Nature.

[25] Dai S (2021). Intratumoral CXCL13^+^CD8^+^T cell infiltration determines poor clinical outcomes and immunoevasive contexture in patients with clear cell renal cell carcinoma. J Immunother Cancer.

[26] Jenh CH (2001). Human B cell-attracting chemokine 1 (BCA-1; CXCL13) is an agonist for the human CXCR3 receptor. Cytokine.

[27] Turner MD (2014). Cytokines and chemokines: at the crossroads of cell signalling and inflammatory disease. Biochim Biophys Acta.

[28] Hao Y (2021). Integrated analysis of multimodal single-cell data. Cell.

[29] Stoeckius M (2017). Simultaneous epitope and transcriptome measurement in single cells. Nat Methods.

[30] Trapnell C (2014). The dynamics and regulators of cell fate decisions are revealed by pseudotemporal ordering of single cells. Nat Biotechnol.

[31] Bardi G (2001). The T cell chemokine receptor CCR7 is internalized on stimulation with ELC, but not with SLC. Eur J Immunol.

[32] Korsunsky I (2022). Cross-tissue, single-cell stromal atlas identifies shared pathological fibroblast phenotypes in four chronic inflammatory diseases. Med.

[33] Mitamura Y (2023). Spatial transcriptomics combined with single-cell RNA-sequencing unravels the complex inflammatory cell network in atopic dermatitis. Allergy.

[34] Hwang ST (2008). Mycosis fungoides and Sézary syndrome. Lancet.

[35] Picchio MC (2008). CXCL13 is highly produced by Sézary cells and enhances their migratory ability via a synergistic mechanism involving CCL19 and CCL21 chemokines. Cancer Res.

[36] Hamanishi J (2010). Activated local immunity by CC chemokine ligand 19-transduced embryonic endothelial progenitor cells suppresses metastasis of murine ovarian cancer. Stem Cells.

[37] Cheng HW (2018). CCL19-producing fibroblastic stromal cells restrain lung carcinoma growth by promoting local antitumor T-cell responses. J Allergy Clin Immunol.

[38] Pietila TE (2007). Multiple NF-kappaB and IFN regulatory factor family transcription factors regulate CCL19 gene expression in human monocyte-derived dendritic cells. J Immunol.

[39] Lodi G (2004). Lichen planus and hepatitis C virus: a multicentre study of patients with oral lesions and a systematic review. Br J Dermatol.

[40] Tziotzios C (2018). Lichen planus and lichenoid dermatoses: clinical overview and molecular basis. J Am Acad Dermatol.

[41] Stuart T (2019). Comprehensive integration of single-cell data. Cell.

[42] McGinnis CS (2019). DoubletFinder: doublet detection in single-cell RNA sequencing data using artificial nearest neighbors. Cell Syst.

[43] Tirosh I (2016). Single-cell RNA-seq supports a developmental hierarchy in human oligodendroglioma. Nature.

[44] Pei G (2022). deCS: a tool for systematic cell type annotations of single-cell RNA sequencing data among human tissues. Genomics Proteomics Bioinformatics.

[45] Stunnenberg HG (2016). The international human epigenome consortium: a blueprint for scientific collaboration and discovery. Cell.

[46] Monaco G (2019). RNA-seq signatures normalized by mRNA abundance allow absolute deconvolution of human immune cell types. Cell Rep.

[47] Schmiedel BJ (2018). Impact of genetic polymorphisms on human immune cell gene expression. Cell.

[48] Becht E (2018). Dimensionality reduction for visualizing single-cell data using UMAP. Nat Biotechnol.

[50] Jin S (2021). Inference and analysis of cell-cell communication using CellChat. Nat Commun.

[51] Qiu X (2017). Reversed graph embedding resolves complex single-cell trajectories. Nat Methods.

