# Supplementary material for: CXCL9, CXCL10, and CCL19 synergistically recruit T lymphocytes to skin in lichen planus
Source: JCI Insight. 2024 Oct 22;9(20):e179899. doi: 10.1172/jci.insight.179899 (PMC11533982; doi:10.1172/jci.insight.179899)
Supplement: Supplemental data [file jciinsight-9-179899-s006.pdf]

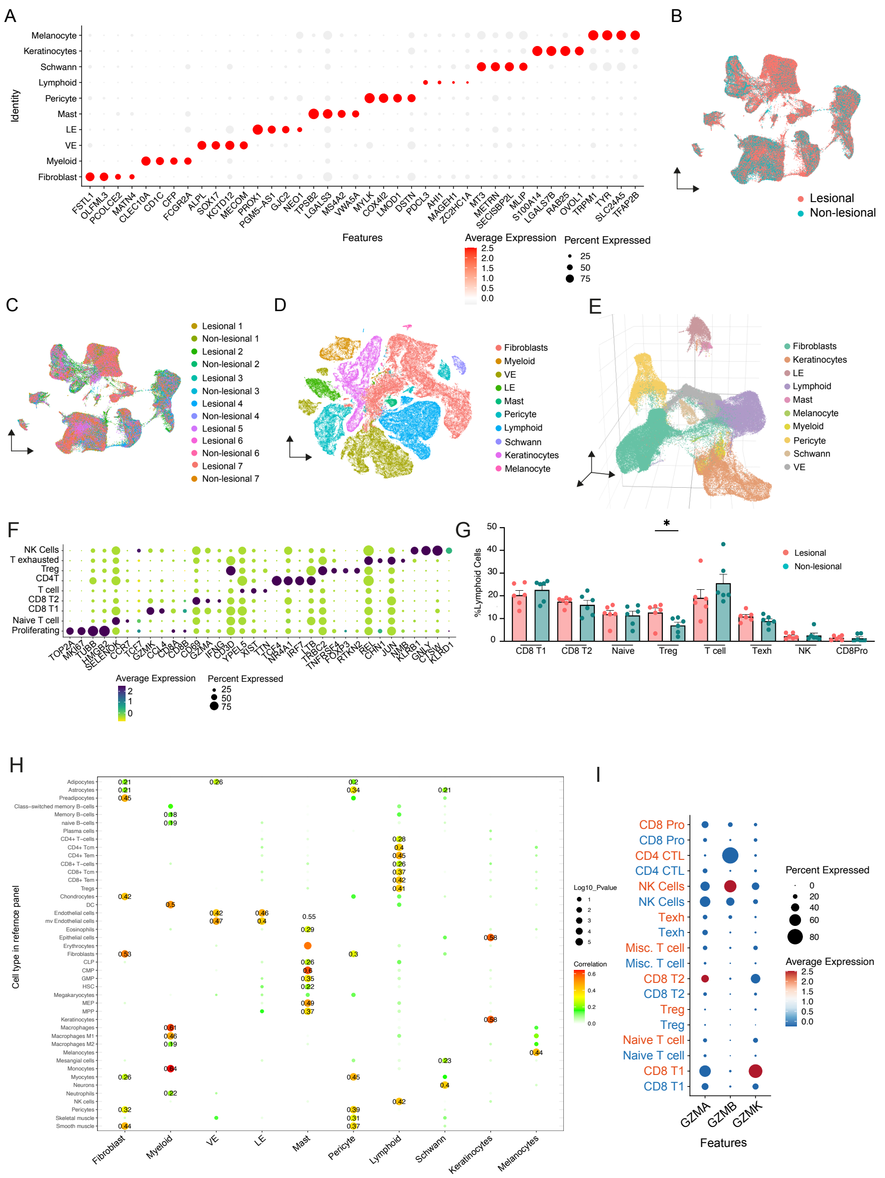

Figure S1: Single cell analysis of lichen planus skin.
(**A**) Dot plot of key marker genes for each cell type. Color scale represents gene expression, and dot size represents percentage of cells expressing the marker gene. (**B and C**) UMAP plots of lesional and non-lesional lichen planus (LP) skin (n=13). Individual patient samples are depicted in C. (**D**) tSNE and (**E**) 3D UMAP depictions of single-cell clusters. (**F**) Dot plot of key marker genes for each lymphoid subcluster. (**G**) Frequency of each lymphoid cell population plotted as a percentage of total skin-infiltrating lymphoid cells. One-way ANOVA, *p<0.05. (**H**) deCS correlation plot for cell type enrichment analysis of different cell clusters. Y-axis: main cell types identified from BlueprintEncode database. Color scale represents Pearson correlation coefficient, and dot sizes represent the -log10 transformed p value. (**I**) Expression of *GZMA, GZMB and GZMK* in lymphoid cells from non-lesional and lesional LP skin. Color represents fold change and size of dot represents number of cells expressing the gene.

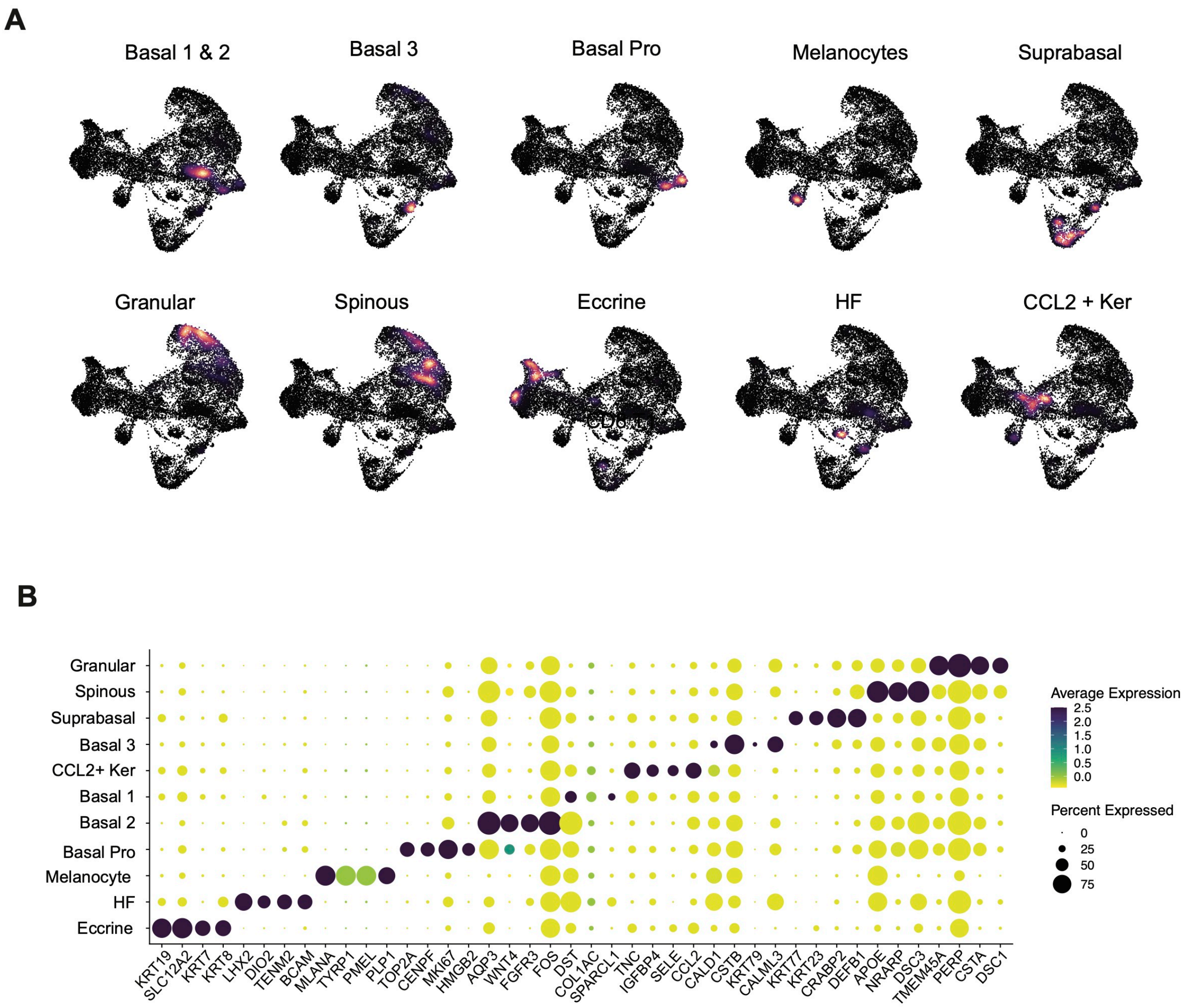

Figure S2: Identification of epidermal cell subclusters.
(**A**) Analysis of individual subclusters. Density plots demonstrates location of subgroup within UMAP. (**B**) Dot plot of key marker genes for each cell type within the epidermis. Color scale represents gene expression and dot size represents percentage of cells expressing the marker gene.

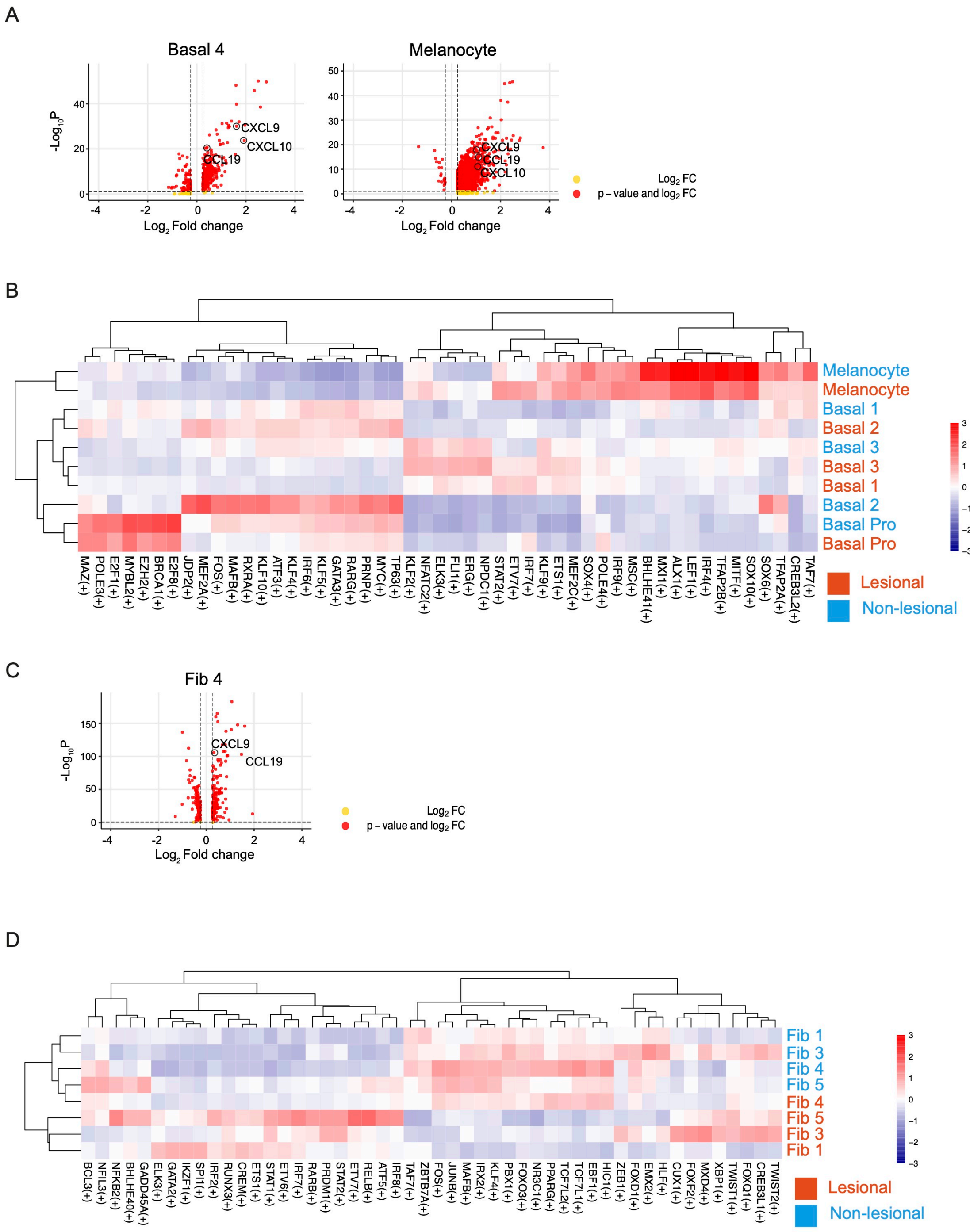

Figure S3: Keratinocyte and fibroblast specific gene expression profiles in lichen planus skin.

(**A**) Differential gene expression of basal keratinocyte cluster #4 and melanocytes in lichen planus (LP) lesional and non-lesional skin highlights expression of *CXCL9*, *CXCL10* and *CCL19*. (**B**) Heatmap of prominent transcription factor modules in LP lesional and non-lesional keratinocyte clusters. (**C**) Differential gene expression of fibroblast cluster 4 in LP lesional and non-lesional skin highlights the expression of *CXCL9* and *CCL19*. (**D**) Heatmap of prominent transcription factor modules in lesional and non-lesional LP fibroblast clusters.

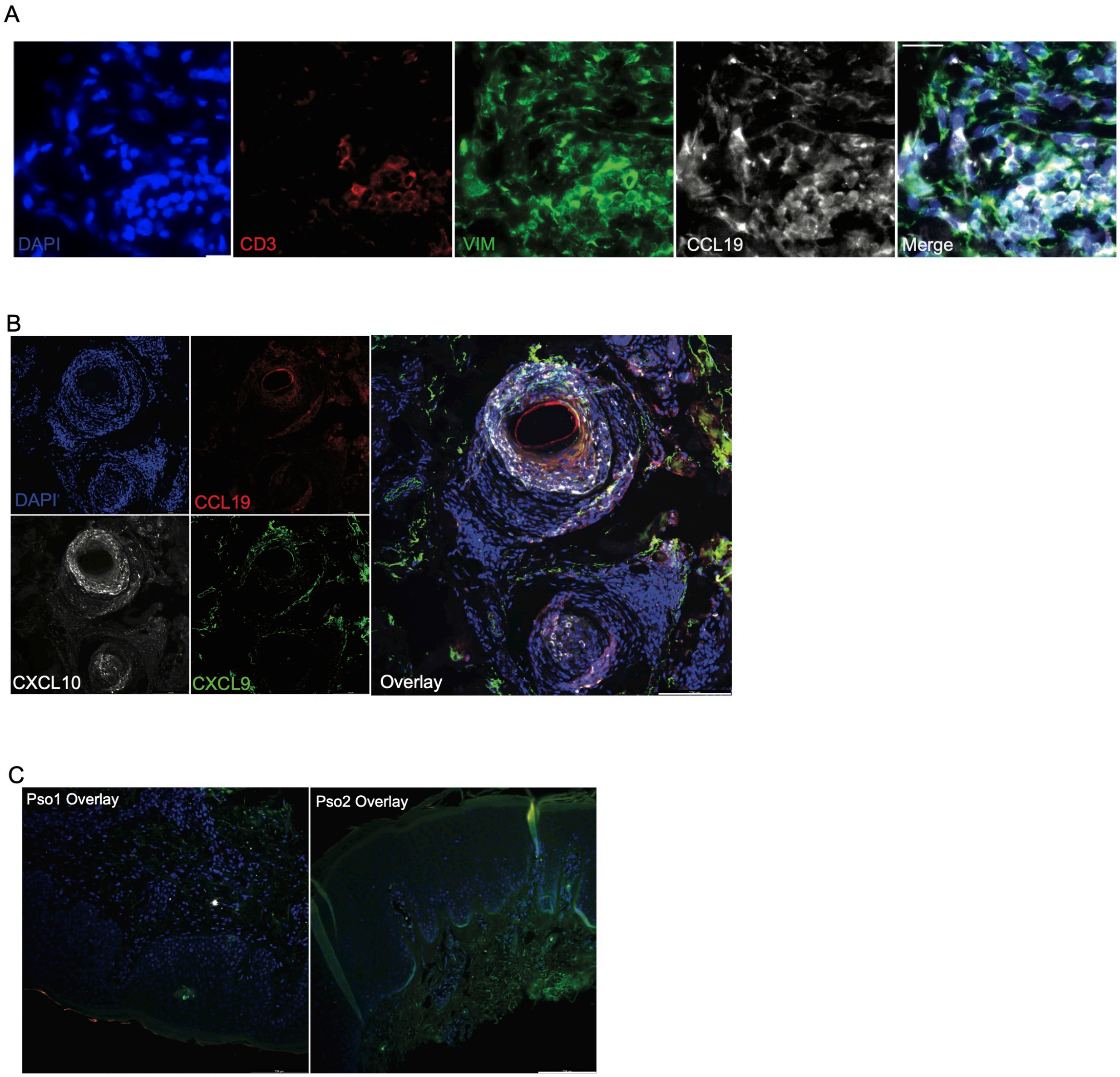

Figure S4: CCL19 staining in lichen planus, lichen planopilaris and psoriasis skin. (**A**) Representative immunofluorescence images depicting CCL19+ fibroblasts in LP skin. CCL19 (white), CD3 (red), vimentin (green), and DAPI (blue) (n=4). (**B**) Representative immunofluorescence images depicting expression of CXCL9, CXCL10, and CCL19 in hair follicle epithelium in lichen planopilaris patients (n=3). CCL19 (red), CXCL10 (white), CXCL9 (green) and DAPI (blue). (**C**) Representative immunofluorescence images depicting absence of CXCL9, CXCL10, and CCL19 in psoriasis skin (n=2). CCL19 (red), CXCL10 (white), CXCL9 (green) and DAPI (blue). Scale bars: 100 micrometer.

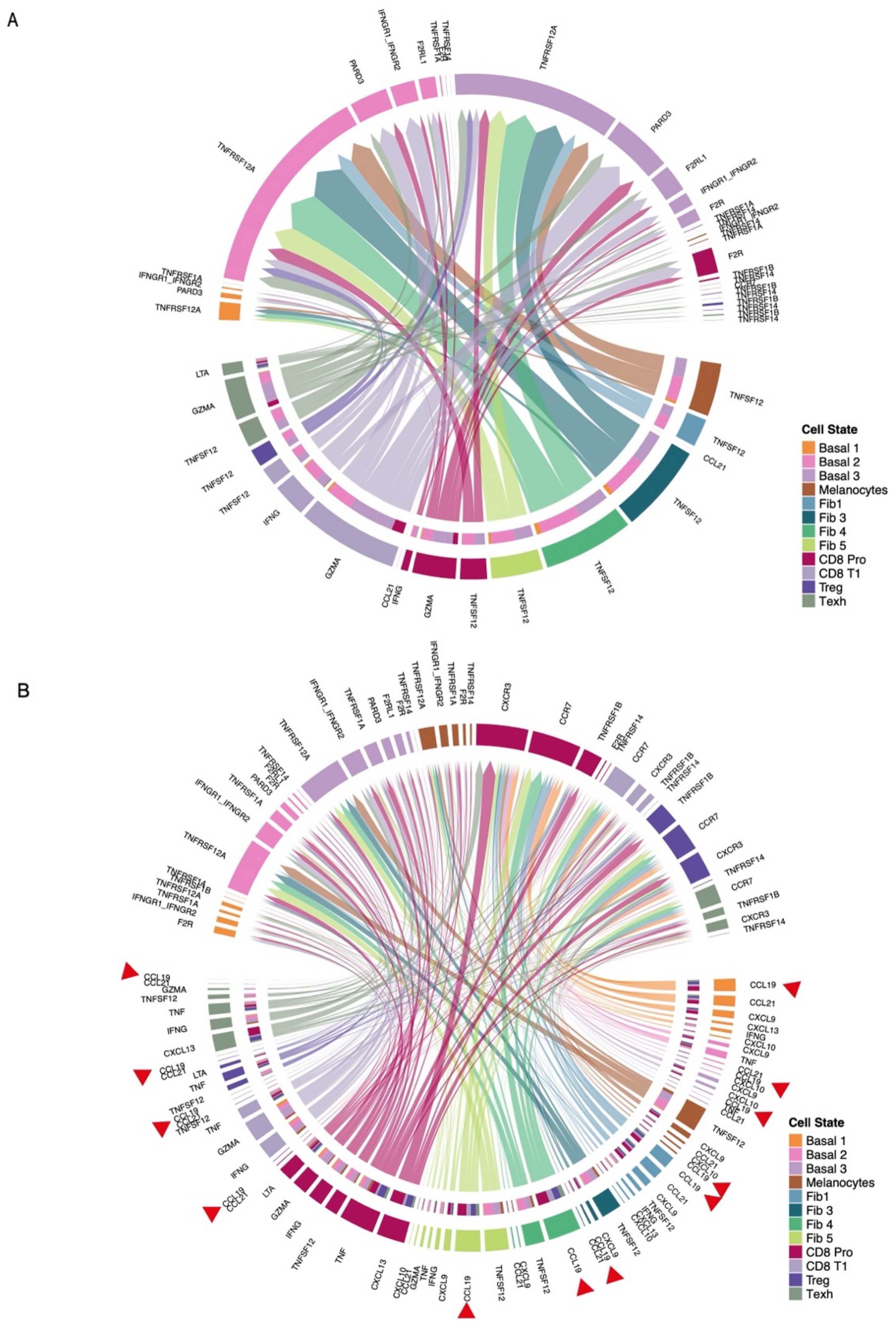

Figure S5: Ligand receptor analysis of lichen planus skin.
(**A-B**) Cell-specific ligand-receptor analysis for non-lesional (**A**) and lesional (**B**) lichen planus skin. Bottom half of the circle depicts secreting cell types. Top half of the circle depicts receiving cell types. The inner bottom-half circle summarizes the receiving cell types by color. Red arrows highlight the majority of CCL19 signal comes from fibroblasts to T cells.

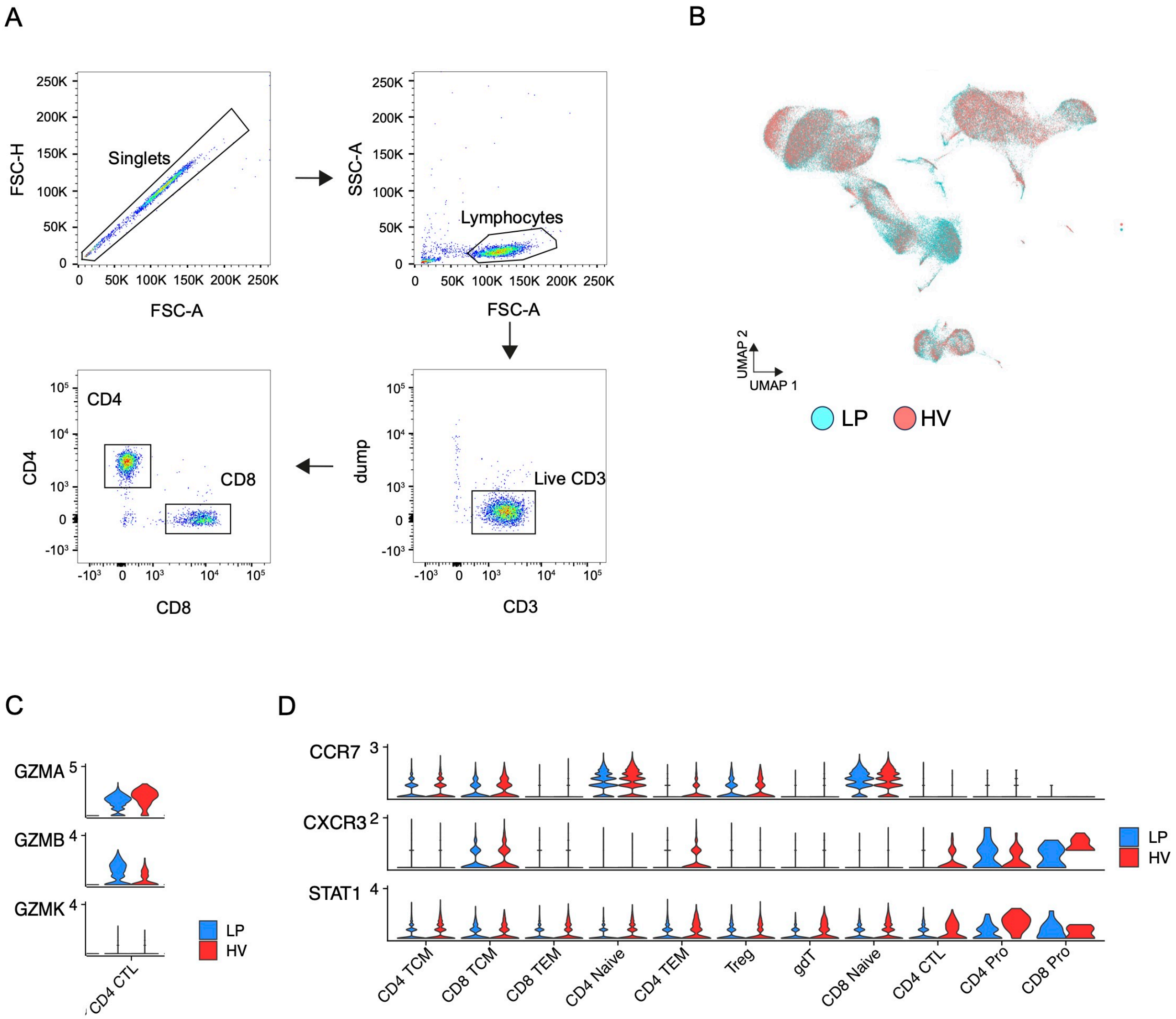

Figure S6: Migration assay and single-cell analysis of lichen planus blood.
(**A**) Flow cytometry gating strategy used to identify CD4 and CD8 subtypes in peripheral blood mononuclear cells (PBMCs). (**B**) UMAP plots of PBMCs from healthy (HV) and lichen planus patients (LP) (n=7). (**C-D**) Violin plot demonstrating average expression *GZMK*, *GZMA*, *GZMB*, *CCR7*, *CXCR3* and *STAT1* in PBMCs from healthy volunteers (HV) and LP patients.

Supplementary Table 1: Patient demographics and clinical information.

	**Patient 01**	**Patient 02**	**Patient 03**	**Patient 04**	**Patient 05**	**Patient 06**	**Patient 07**
**Age (years)**	75	62	43	54	39	63	62
**Sex**	M	M	F	F	M	F	F
**Race/ Ethnicity**	C - Hispanic/Latino	C	C	C	C	C	C
**Histology**	Y	Y	Y	Y	Y	Y	Y
**Sites affected**	Skin	Skin	Skin	Skin, mucosa (oral and vaginal)	Skin, scalp/hair, nails, mucosa (oral)	Skin	Skin, mucosa (oral, vaginal, esophageal)
**Disease duration**	7 months	1 year	2 months	7 years	4 months	2 years	10 years
**Treatment(s) at time of recruitment**	None	None x1 week (clobetasol PRN)	Prednisone 30 mg (6 days)	Rare tacrolimus ointment	None	HCQ, topical tacrolimus (mucosa only)	Dexamethasone elixir (swish and spit)
**Prior treatment(s)**	Triple paste	None	None	HCQ, topical steroids, ILK	Oral steroid (short course)	Doxycycline, metronidazole, topical steroids	
**Lesional Biopsy location**	Left clavicle medial	Right wrist	Right shoulder-inferior	Left shin	Left hip inferior	Right thigh lateral	Upper back lateral
**Nonlesional biopsy location**	Left clavicle lateral	Right forearm	Right shoulder-superior	Left medial lower leg	Left hip superior	Right thigh medial	Upper back medial
**Blood**	Y	Y	Y	Y	Y	Y	Y

C, Caucasian. Y, yes. PRN, as needed. HCQ, hydroxychloroquine. ILK, intralesional Kenalog.

Supplemental Table 2: Sample details with sequencing statistics for single cell RNAseq data.

Disease	Patient ID	Sample ID	Sequencing library ID	Tissue	Median filtered genes per cell	Median filtered UMIs per cell	Median value of mitoRatio	Median filtered number of cells
LP	Patient 01	Affected 01	LP1	Skin	1122	2638	0.04	23436
LP	Patient 01	Unaffected 01	LC1	Skin	1660	4062	0.05	7870
LP	Patient 02	Affected 02	LP2	Skin	823	1577	0.04	15110
LP	Patient 02	Unaffected 02	LC2	Skin	670	1160	0.04	13393
LP	Patient 03	Affected 03	LP3	Skin	1714	4182	0.05	3961
LP	Patient 03	Unaffected 03	LC3	Skin	1278	2984	0.05	7649
LP	Patient 04	Affected 04	LP4	Skin	1877	4492	0.03	14180
LP	Patient 04	Unaffected 04	LC4	Skin	2468	7181	0.04	2545
LP	Patient 05	Affected 05	LP5	Skin	1678	3221	0.04	7924
LP	Patient 06	Affected 06	LP6	Skin	1464	4025	0.05	8810
LP	Patient 06	Unaffected 06	LC6	Skin	2681	8704	0.03	5247
LP	Patient 07	Affected 07	LP7	Skin	2137	6125	0.07	6268
LP	Patient 07	Unaffected 07	LC7	Skin	2261	6452	0.05	6129
LP	Patient 01	LPBMC1	LPBMC1	Blood	1320	3569	0.05	20761
LP	Patient 02	LPBMC2	LPBMC2	Blood	378	601	0.07	3213
LP	Patient 03	LPBMC3	LPBMC3	Blood	1506	4419	0.05	892
LP	Patient 04	LPBMC4	LPBMC4	Blood	1532	4427	0.06	8920
LP	Patient 05	LPBMC5	LPBMC5	Blood	1365	3940	0.06	12599
LP	Patient 06	LPBMC6	LPBMC6	Blood	1728	5738	0.06	7780
LP	Patient 06	LPBMC7	LPBMC7	Blood	1612	4964	0.05	12190
	TOTAL							188607

UMIs, unique molecular identifiers. LP, lichen planus.
